# Partial Response After Toripalimab Plus Anlotinib for Advanced Metaplastic Breast Carcinoma: A Case Report

**DOI:** 10.3389/fendo.2022.810747

**Published:** 2022-03-23

**Authors:** Yang Fu, Jie Liu, Yu Jiang

**Affiliations:** Department of Medical Oncology, Cancer Centre, West China Hospital, Sichuan University, Chengdu, China

**Keywords:** metaplastic breast carcinoma, immunotherapy, anti-angiogenic, outcome, PR

## Abstract

Metaplastic breast carcinoma (MBC) is an aggressive subtype of breast cancer, accounting for <1%. The clinical outcome is unknown due to the lack of treatment options. Here, we present the case of a 58-year-old woman with advanced MBC, in which standard adjuvant chemotherapy was unsuccessful. In the second-line therapy, she received anti-angiogenic(anlotinib) therapy plus chemotherapy. Finally, she was subsequently treated with immunotherapy (toripalimab) combined anlotinib and achieved partial response (PR); thus, immunotherapy plus anti-angiogenic therapy might be a novel option for advanced MBC patients.

## Introduction

Metaplastic breast carcinoma (MBC) is a rare and aggressive subtype of breast cancer (about 1%) (1). MBC occurs commonly in women over the age of 60 years, typically presenting as a larger tumor size (2, 3). According to the WHO breast tumor classification in 2019, MBC was divided into five subtypes including adenosquamous carcinoma, pure squamous cell carcinomas, pure spindle cell carcinoma, metaplastic carcinoma with mesenchymal differentiation, and mixed metaplastic carcinoma (1). More than 80% of MBC did not express estrogen receptor (ER), progesterone receptor (PR), and human epidermal growth factor 2 receptor (HER2) (4). Advanced MBC has a poor prognosis compared to non-MBC triple-negative breast cancer (TNBC) due to rapid tumor growth and insensitivity to standard chemotherapy (5, 6). Immune checkpoint inhibitors (ICIs) block the PD-L1/PD-1 and CTLA-4/B7 signaling pathways, thereby preventing effector T cells from being inactivated and maintaining/keeping them to be able to kill tumor cells. In the past decade, immunotherapy improved survival benefits for patients with TNBC; whether immunotherapy is effective for MBC is still unknown (7–12). Here, we report an advanced MBC patient who failed with standard chemotherapy in the first-line therapy and anlotinib plus chemotherapy in the second-line therapy. She was subsequently treated with toripalimab plus anlotinib and achieved partial response (PR). Thus, immunotherapy combined with anti-angiogenic therapy might be a novel option for advanced MBC patients in later-line treatment.

## Case Report

**Figure 1:**
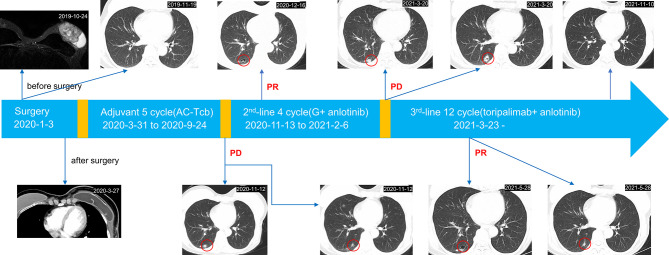
Complete treatment process of the patient since diagnosis.

**Figure 2:**
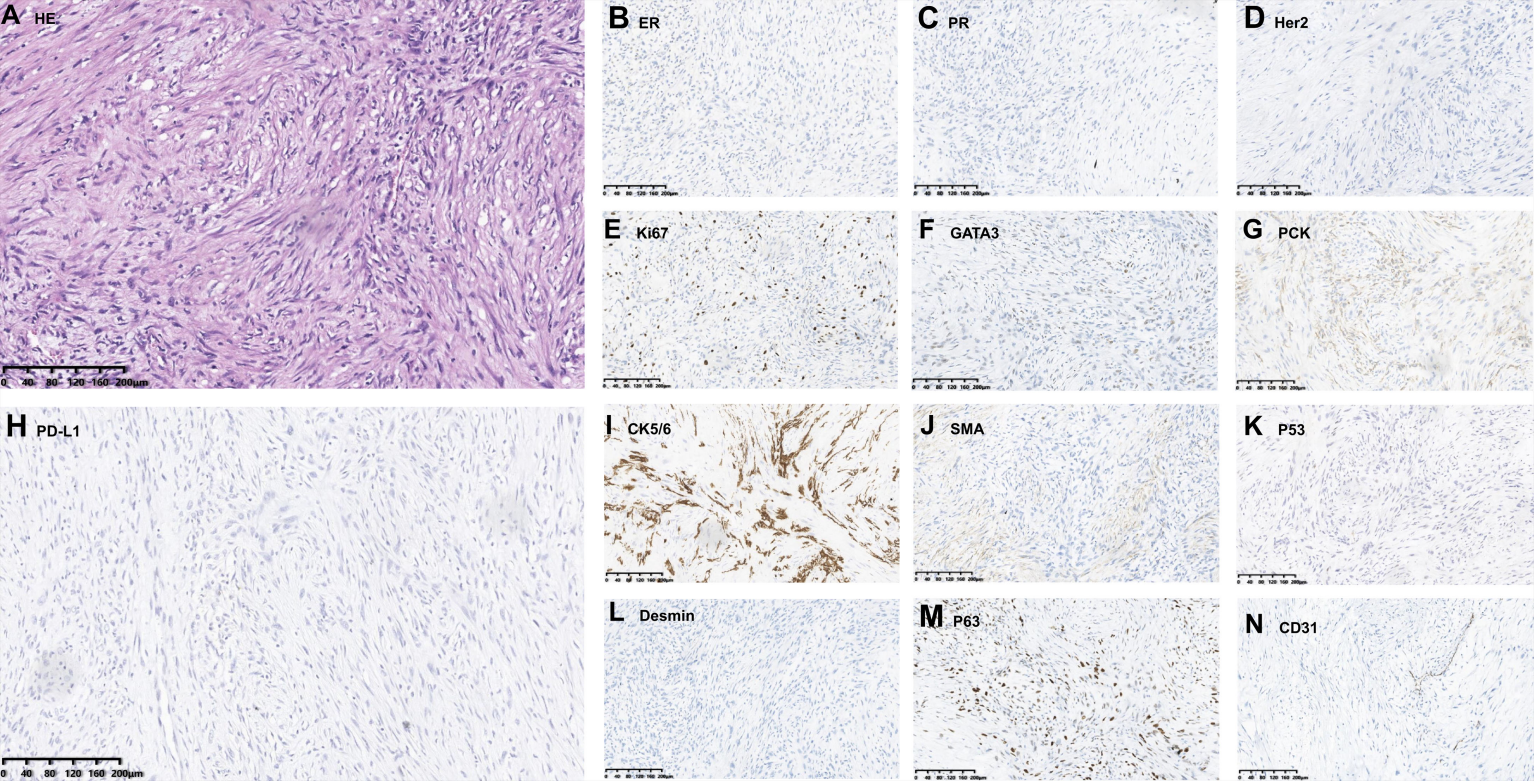
Pathological examination showed spindle cell morphology (**A**, H&E). **(B–N)** Immunohistochemistry data: ER (−), PR (−), Her2(−), Ki-67 (+, positive proportion about 20%), GATA3 (+), PCK (+), PD-L1(−, positive proportion about <1%), CD5/6 (+), SMA (+), P53(−), Desmin (−), P63(+), and CD31 (−) supported the diagnosis. Original magnification: **(A–N)**, 200×.

A 58-year-old female came to our hospital with a chief complaint of finding a left breast mass. Breast ultrasound showed a left BI-RADs 4c breast mass and enlarged left axillary lymph nodes (**Figure 1**). The tumor biomarkers such as carcinoembryonic antigen (CEA) (1.2 ng/ml) and cancer antigen 153 (CA 153) (8.63 U/ml) were in the normal range. She underwent modified radical mastectomy, axillary lymph node dissection, and breast reconstruction. MBC (squamous cell carcinoma and sarcomatoid components) was established by pathological examination and confirmed by immunohistochemistry (IHC) staining, which demonstrated ER (−), PR (−), Her2 (0), Ki67(+, 20%), GATA3 (+), PCK (+), P63 (+), CK5/6 (+), SMA (+), P53(−), Desmin (−), Myogenin (−), and STAB2 (−). CD31 stain was negative in the tumor cell and positive in the tumor vasculature. PD-L1 expression in the tumor cell and tumor vasculature was assessed using antibody 22C3 (Agilent Technologies, USA) and a combined positive score of <1% (**Figure 2**). All of the three excised lymph nodes were free of tumor cells (T3N0M0, stage IIB). Adjuvant chemotherapy was prescribed after surgery, and chemotherapy regimen consisted of anthracycline plus cyclophosphamide followed by paclitaxel plus carboplatin (AC-TCb). After four cycles of AC and one cycle of TCb, multiple pulmonary metastases (>5, **Supplementary Figure S1** in the **Supplementary Appendix**) in the lung were shown in the following chest CT scan. The efficacy was evaluated as progression disease (PD). Anlotinib (10 mg, qd, days 1–14) combined with gemcitabine (1,400 mg, every 3 weeks) was prescribed for the second-line therapy. After two cycles of combined therapy, the metastases in the lung achieved PR, while after four cycles, we rechecked the enhanced CT images, and the efficacy evaluation was PD (**Supplementary Figure S2**). After multi-disciplinary treatment, we changed the original scheme, and toripalimab (an anti-PD1 antibody, Junshi Inc., China, Shanghai) at a dose of 160 mg(3 mg/kg) combined with anlotinib (10 mg, qd, days 1–14) was given every 3 weeks. After two cycles of combined therapy, the size of pulmonary metastases became smaller with no treatment-related adverse events. Up to now, the patient sustained remission more than 8 months without further complaints and side effects (sustained PR) and continue to receive anlotinib plus toripalimab regularly.

## Discussion

MBC is a rare and aggressive subtype of breast cancer. Reddy et al. showed that MBC has a lower OS than non-MBC (64.4 vs. 159.2 m, *p* < 0.001) (13). No standard of care for this disease is established, while the current therapy often contains chemotherapy. Our patients received immunotherapy plus anti-angiogenic therapy, which has been shown to improve the prognosis of MBC.

Previous studies showed that MBC had a unique tumor environment. Several studies observed a high level of PD-L1 expression and high density of CD8+ tumor infiltrating lymphocytes (TILs) in this tumor. Upasana et al. showed that MBC has the highest PD-L1 expression and CD8+ TILs than the other subtypes of breast cancer (14). Lien et al. observed that the positivity for TILs, combined positive score (CPS), and tumor proportion score (TPS) were 34.1%, 47.6%, and 17.1%, respectively. In addition, squamous cell carcinoma components in MBC had the highest positivity rates of TILs and CPS (15). Kalaw et al. enrolled 146 MBC patients, and 73% of them had PD-L1 expression in tumor ≥5% (16). In addition, Joneja et al. tested the expression of PD-L1 in 72 MBC patients, and the results showed positive PD-L1 of tumor and immune cells at 46% and 43%, respectively (14). High PD-L1 expression and TILs are generally associated with good response to ICIs.

ICIs have changed the treatment landscape of advanced breast cancer. Impassion 130 was a randomized phase III study that tested the efficiency of atezolizumab combined with nab-paclitaxel in the first-line treatment. The chemo-immunotherapy showed benefit in both median progression-free survival (PFS) (7.5 m vs. 5.3 m, *p*<0.001) and OS (21.0 m vs. 18.7 m, *p* = 0.078) compared with placebo plus nab-paclitaxel (8). A similar good outcome was also achieved by pembrolizumab. In the phase III KEYNOTE-355 study, the combination of pembrolizumab and chemotherapy achieved an mPFS of 9.7m (CPS > 10) (7). In a single-arm, phase 2 trial (DART trial), 17 MBC patients received the nivolumab and ipilimumab combination therapy. The ORR, median PFS, and median OS were 18%, 2 months, and 12 months, respectively (17). Previously, four case reports observed the efficacy of ICIs in advanced MBC (**Table 1**). Six of eight patients showed a good prognosis after immunotherapy (18–21).

**Table 1:** Summary of case reports observing the efficacy of ICI in MBC.

Source	Age (y)	Histology	ER	PR	HER2	PD-L1	Line	Therapy	PFS (m)	Response	OS (m)	Death
Adams (18)	53	Spindle cell carcinoma	–	–	–	100%	3rd	Pembrolizumab+ nab-paclitaxel	6	PR	>6	No
Sayed et al. (19)	49	Squamous cell carcinomas	–	–	+	20%	4th	Durvalumab+ paclitaxel	>24	PR	>24	No
Gorshein et al. (20)	72	Mixed metaplastic carcinoma	–	–	–	positive	1st	Pembrolizumab	24	PR	32	No
Kim et al. (21)	63	Metaplastic squamous carcinoma	–	–	–	0%	1st	Pembrolizumab+ capecitabine	6	PR	>6	No
	58	Metaplastic carcinoma mesenchymal	–	–	–	0%	1st	Pembrolizumab+ capecitabine	6	PR	NR	No
	82	Mixed metaplastic carcinoma	+	+	–	30%	3rd	Nivolumab+ bicalutamide	8	CR	NR	NR
	60	Metaplastic squamous carcinoma	–	–	–	10%	1st	Pembrolizumab+ capecitabine	3	PD	NR	NR
	62	Metaplastic carcinoma mesenchymal	–	–	–	0%	1st	Pembrolizumab+ paclitaxel	NR	PD	NR	Yes

NR, not reported; CR, complete response; PR, partial response; PD, progressive disease; m, months; y, years.

Anlotinib is a Chinese multitarget tyrosine kinase inhibitor (TKI), which can inhibit VEGFR1, VEGFR2, VEGFR3, c-Kit, and PDGFR, and is approved by the Chinese National Medical Products Administration for the treatment of advanced non-small-cell lung carcinoma (NSCLC), SCLC, soft-tissue sarcoma, and medullary thyroid carcinoma in the later line (22–25). Hu et al. investigate anlotinib for HER2-negative breast cancer in later-line therapy. The mPFS was 5.22 m, and the disease control rate (DCR) was 80.8%. Meanwhile, severe adverse events (AEs, ≥G3) were hypertension (26.92%) and hand–foot syndrome (3.85%). These results showed that anlotinib had good efficacy and limited toxicity with HER2-negative breast cancer (26). Only one case of advanced MBC treated with anlotinib has been reported. This MBC patient underwent anlotinib (12 mg/day, 2 weeks on, 1 week off), and achieved a durable PR for more than 25 months (27).

Several studies demonstrated that anti-angiogenic agents have synergistic effects with ICIs. Antiangiogenic therapy can make abnormal tumor vessels normalization, which increases the infiltration of immune effector cells in TME (28). In three phase 3 trials (IMBrave150, KEYNOTE-426, and IMpower150), atezolizumab combined with bevacizumab, pembrolizumab combined with axitinib, and atezolizumab combined with bevacizumab+ chemotherapy were shown to bring survival benefit to advanced hepatocellular carcinoma, advanced renal cell carcinoma, and advanced NSCLC, respectively (29–32). No case report described the efficacy of these combination therapy in advanced MBC. However, there were no clinical studies that reported the anlotinib plus ICI in earlier line treatment for MBC. We look forward to observing prospective clinical trials to explore the efficacy of the combined scheme on MBC in the future. In addition, it is necessary to explore the relationship of PD-L1 expression and vascularization for the efficacy of anlotinib and toripalimab.

In conclusion, we described a case of advanced MBC treated with toripalimab plus anlotinib after failure of standard chemotherapy and chemotherapy plus anti-angiogenic therapy. Immuno-combined anti-angiogenic therapy might be a useful candidate for advanced MBC.

## Data Availability Statement

The original contributions presented in the study are included in the article/**Supplementary Material**. Further inquiries can be directed to the corresponding author. The full original source data can access in https://www.jianguoyun.com/p/DcmPFKQQ7oeDChiYyJsE.

## Ethics Statement

The studies involving human participants were reviewed and approved by Ethics Committee on Biomedical Research, West China Hospital of Sichuan University. The patients/participants provided their written informed consent to participate in this study. Written informed consent was obtained from the individual(s) for the publication of any potentially identifiable images or data included in this article.

## Author Contributions

YJ and JL contributed to conception of the study. YF drafted the manuscript. YJ reviewed the manuscript. JL edited the manuscript. All authors contributed to the article and approved the submitted version.

## Conflict of Interest

The authors declare that the research was conducted in the absence of any commercial or financial relationships that could be construed as a potential conflict of interest.

## Publisher’s Note

All claims expressed in this article are solely those of the authors and do not necessarily represent those of their affiliated organizations, or those of the publisher, the editors and the reviewers. Any product that may be evaluated in this article, or claim that may be made by its manufacturer, is not guaranteed or endorsed by the publisher.

## Supplementary Material

Supplementary Figure 1 The CT showed that mutiple metastases of lung after adjuvant chemotherapy.

Supplementary Figure 2 The CT showed that multiple pulmonary metastases after anlotinib plus gemcitabine.
