# Supplementary figures and images for: Partial Response After Toripalimab Plus Anlotinib for Advanced Metaplastic Breast Carcinoma: A Case Report
Source: Front Endocrinol (Lausanne). 2022 Mar 23;13:810747. doi: 10.3389/fendo.2022.810747 (PMC8984491; doi:10.3389/fendo.2022.810747)

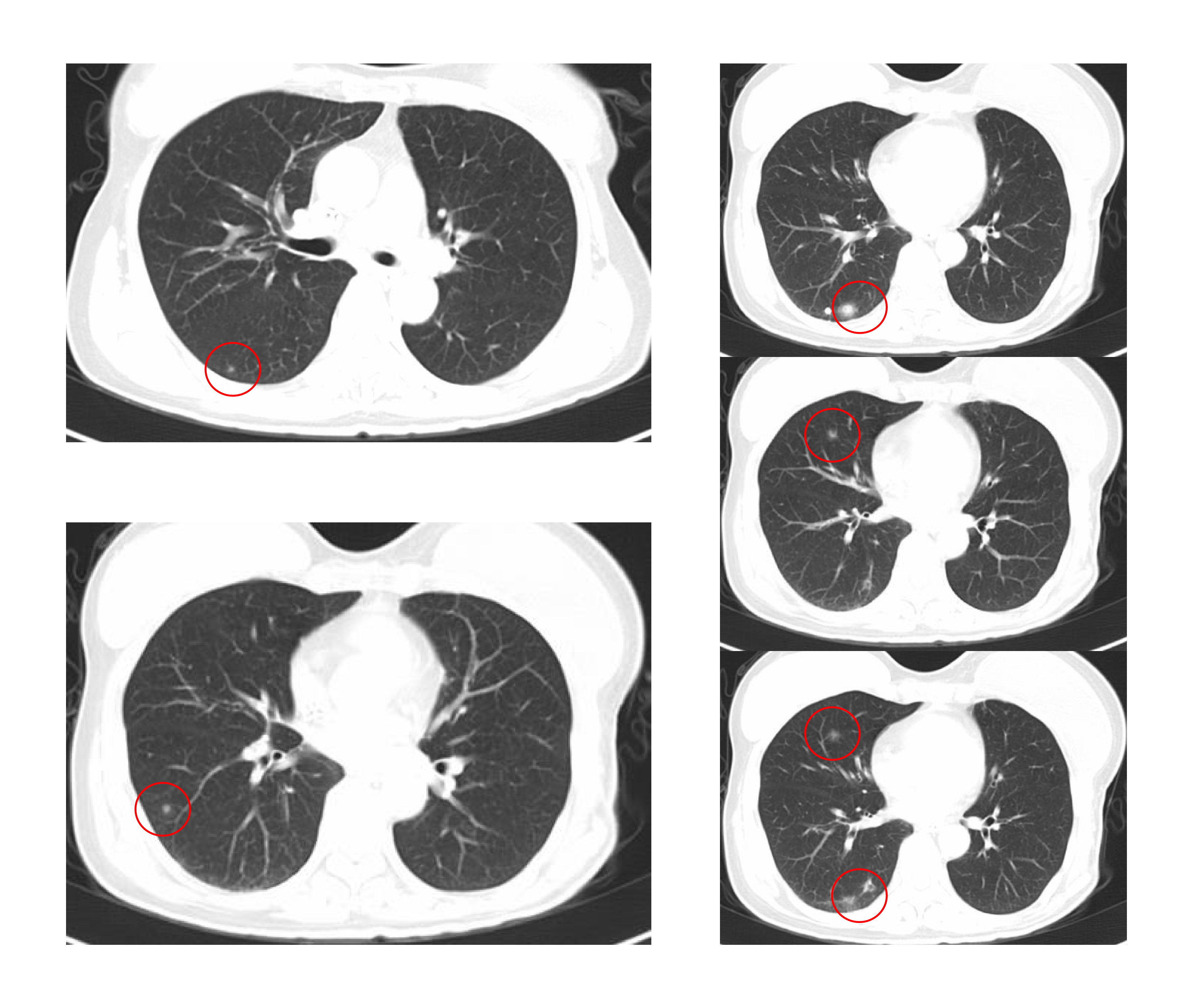

Supplement: Supplementary Figure 1 — The CT showed that mutiple metastases of lung after adjuvant chemotherapy. [file Image_1.tiff]

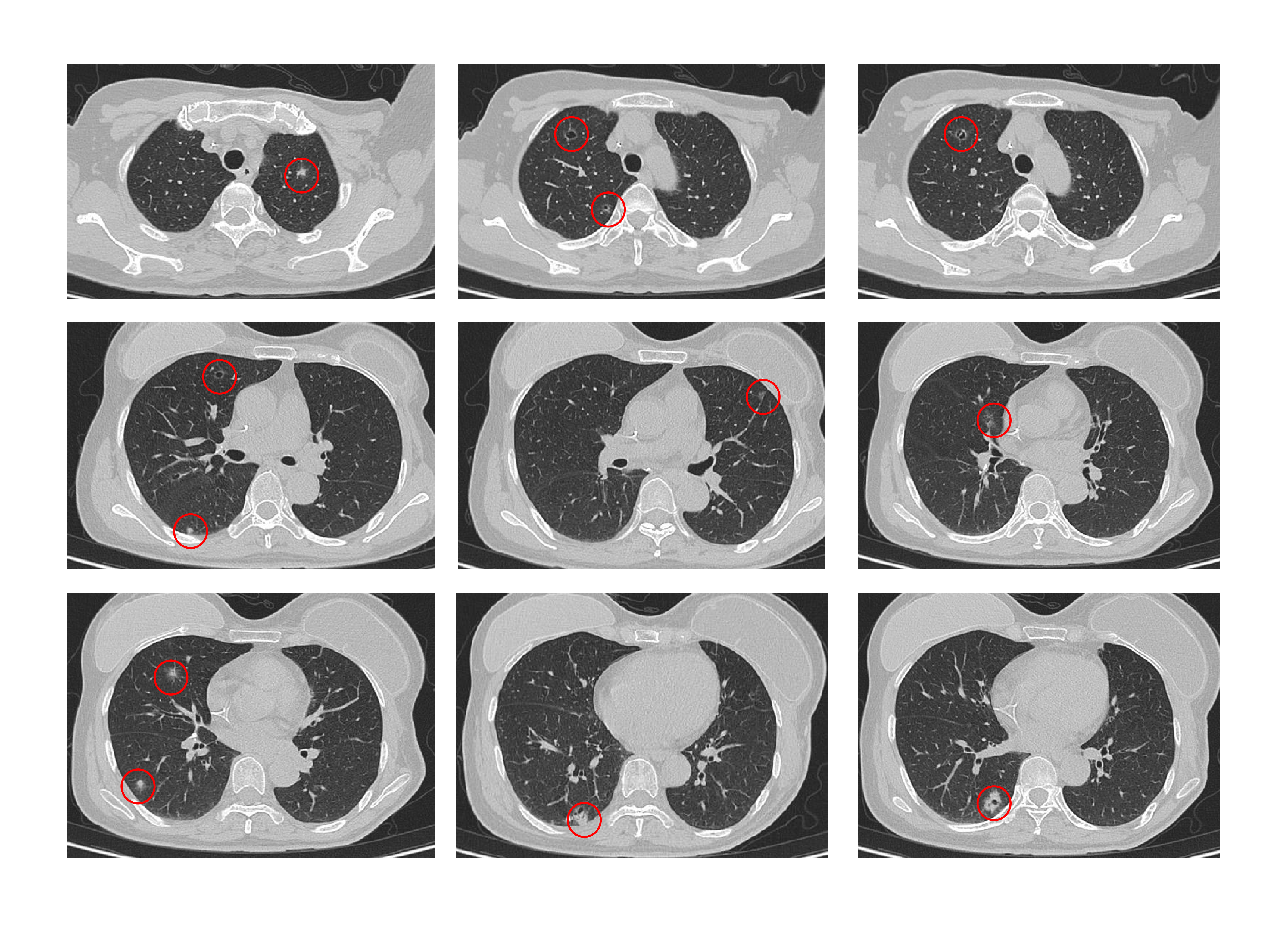

Supplement: Supplementary Figure 2 — The CT showed that multiple pulmonary metastases after anlotinib plus gemcitabine. [file Image_2.tiff]
